# Icariin-Curcumol promotes docetaxel sensitivity in prostate cancer through modulation of the PI3K-Akt signaling pathway and the Warburg effect

**DOI:** 10.1186/s12935-023-03042-1

**Published:** 2023-09-02

**Authors:** Wenjing Xu, Jin Ding, Shida Kuang, Bonan Li, Tiansong Sun, Congxu Zhu, Juan Liu, Lemei Zhu, Yingqiu Li, Wen Sheng

**Affiliations:** 1 Department of Dermatology, The First Affiliated Hospital of Hunan University of Chinese Medicine, Changsha, 410021 China; 2 Department of Andrology, Shenzhen Bao’an Traditional Chinese Medicine Hospital, Guangzhou University of Chinese Medicine, Shenzhen, 518133 China; 3 Andrology Laboratory, Hunan University of Chinese Medicine, Changsha, 410208 China; 4 School of Traditional Chinese Medicine, Hunan University of Chinese Medicine, Changsha, 410208 China; 5 School of Integrated Chinese and Western Medicine, Hunan University of Chinese Medicine, Changsha, 410208 China; 6 School of Public Health, Changsha Medical University, Changsha, 410219 China; 7 Academician Workstation, Changsha Medical University, Changsha, 410219 China; 8 Medical School, Hunan University of Chinese Medicine, Changsha, 410208 China

**Keywords:** Prostate cancer, Docetaxel, PI3K-Akt signaling pathway, Warburg effect, Icariin, Curcumol

## Abstract

**Background:**

Docetaxel (DTX) resistance reduces therapeutic efficacy in prostate cancer (PCa). Accumulating reports support the role of phytochemicals in the reversal of DTX resistance. This study aimed to determine whether *Epimedium brevicornu* and *Curcuma zedoaria* extracts (ECe), specially icariin-curcumol, attenuates DTX resistance and explore their potential mechanisms.

**Methods:**

Regulatory pathways were predicted between ECe active ingredients and PCa using network pharmacology. DTX-resistant cell LNCaP/R were established based on DTX-sensitive LNCaP, and xenograft models were further established. Active ingredients in ECe by HLPC-MS were identified. The binding of icariin and curcumol to the target was analyzed by molecular docking. Biochemical experiments were applied to determine the possible mechanisms by which Icariin-Curcumol regulates DTX sensitivity.

**Results:**

Akt1 and the PI3K-Akt signaling pathway were predicted as the primary functional target between drug and PCa. ECe and DTX inhibited xenograft tumor growth, inflammation, cell viability and promoted apoptosis. Icariin and curcumol were detected in ECe, and icariin and curcumol docked with Akt1. ECe, Icariin-Curcumol and DTX downregulated AR, PSA, PI3K, Akt1, mTOR, and HIF-1ɑ. Moreover, ECe, Icariin-Curcumol and DTX increased glucose and PDH, decreased lactic acid, ATP and LDH, and downregulated c-Myc, hnRNPs, VEGF, PFK1, and PKM2. Notably, the anti-PCa effect of DTX was attenuated compared to ECe or Icariin-Curcumol in the LNCaP/R model. The combined effect of Icariin-Curcumol and DTX was superior to that of DTX.

**Conclusion:**

Our data support that Icariin-Curcumol reverses DTX resistance by inhibiting the PI3K-Akt signaling and the Warburg effect, providing new ideas for improving therapeutic measures for PCa.

## Introduction

Prostate cancer (PCa) is the leading cause of cancer-related deaths in men worldwide. Approximately 20% of PCa patients die each year, seriously endangering men’s health and increasing the burden on society [1, 2]. Currently, androgen deprivation therapy (ADT) is widely accepted as the clinical paradigm for the treatment of advanced PCa and metastatic disease [3]. However, the vast majority of patients who receive ADT inevitably develop castration-resistant prostate cancer (CRPC) [4]. Docetaxel (DTX), a paclitaxel derivative, has been identified as the first-line drug of choice for treating CRPC [5]. Studies have shown that DTX can block vascular depolymerization to induce mitotic arrest and apoptosis in cancer cells [6]. In addition, DTX has been shown to block androgen receptor (AR) translocation to the nucleus and limit AR expression [7]. However, the emergence of DTX resistance has led to less favorable survival rates in CRPC patients [8]. Therefore, it is of great significance to explore the mechanisms of DTX resistance.

*Epimedium brevicornu* (EB, Yinyanghuo) is a traditional Chinese herb with outstanding medicinal properties in cancer, neurodegenerative diseases, osteoporosis, and erectile dysfunction due to its main active components, icariin and icariside II [9]. There is evidence that Icariside II exerts anti-inflammatory and apoptosis-inducing effects in PC-3 cells (androgen non-dependent) and involves the restriction of cyclooxygenase-2 (COX2)/prostaglandin E2 (PGE2) [10]. In addition, EB extract enhances AR expression in LNCaP cells (androgen-dependent) and promotes the growth of PC xenograft tumors. Epimedium II was assayed for anti-androgenic activity in LNCaP cells by luciferase assay [11]. Icaristin was reported to inhibit LNCaP cell proliferation and induce apoptosis and cell cycle arrest [12].

*Curcuma zedoaria* (CZ, Ezhu), another traditional Chinese herb, belongs to the genus *Curcuma* and its main components, curcumin and curcumol, have anti-cancer, anti-bacterial, anti-inflammatory and anti-oxidant and other pharmacological activities [13, 14]. The modulatory functions of curcumin in PCa have gradually emerged [15]. Curcumin promotes apoptosis of LNCaP cells and limits the expression of AR and prostate-specific antigen (PSA) [16]. Moreover, curcumol inhibits PC-3 and 22RV1 cell (low androgen-dependent) viability, migration and invasion, promotes apoptosis and impedes tumor growth [17, 18].

Investigative studies by researchers support the efficacy of combination therapies of two or more substances over single-substance treatment modalities [19]. Previous studies have found synergistic effects of icariin and curcumol in regulating the development of PCa [20]. However, few reports have focused on the role of EB-CZ extract (ECe), specifically icariin-curcumol, in combating DTX resistance. Therefore, in this study, we used network pharmacology to analyze the association of EB-CZ active components with molecular networks in PCa and determined the modulation of major predictive pathways by ECe in animal and cell models. Additionally, we discussed the effect of ECe on DTX resistance.

## Methods

### Network pharmacology

The active ingredients and targets of EB and CZ were obtained from the TCMSP database (https://tcmspw.com/tcmsp.php) screening with conditions set to OB ≥ 30% and DL ≥ 0.18. All targets were corrected by the uniprot database (https://www.uniprot.org/) to remove non-human targets and duplicate targets. The keywords “prostate cancer” were used in the GeneCards database (https://www.genecards.org/), NCBI gene database (https://www.ncbi.nlm.nih.gov/) and OMIM database (https://www.omim.org/) to screen out disease-related targets. After aggregating and removing duplicate values, the common values of EB-CZ targets and disease targets were filtered and visualized using a Venn diagram (Venny 2.1 software). Subsequently, the common targets were extracted and entered into the String database (https://string-db.org/cgi/input.pl) to construct a protein-protein interaction (PPI) network, with the biological species set as “Homo sapiens”. The data were imported into Cystoscape 3.8.0 to map the ingredient-target-PC network, and topological analysis was performed by the NetworkAnalyzer tool. Genes with scores greater than the mean score were selected as key targets by degree sorting, and the top 20 targets were visualized by R software 4.0.3. In addition, the common targets were subjected to KEGG enrichment analysis. R software 4.0.3 and clusterProfiler package were applied to visualize the top 20 important pathways by bubble plots. A network diagram of EB-CZ active ingredient-target-functional pathway-PCa was demonstrated using Cystoscape 3.8.0.

### Cell culture

Human prostate cancer LNCaP cells (CL-0143, Procell) were maintained in RPMI-1640 medium supplemented with 10% fetal bovine serum (FBS) and 1% penicillin/streptomycin. DTX-resistant PCa cells were established by sequential exposure to increasing concentrations of DTX, as previously described [21]. Briefly, LNCaP cells were sequentially treated with 0.1 nM, 0.2 nM, 0.5 nM, 1 nM, 5 nM, and 10 nM DTX. Once the cells remained free to grow at 10 nM DTX, the cells were identified as DTX-resistant and labeled as LNCaP/R cells. Sequential concentrations (1 nM, 5 nM, 10 nM, 20 nM, 50 nM) of DTX and DMSO were applied to treat LNCaP and LNCaP/R cells to verify cell resistance.

### Cell counting kit-8 (CCK8) assay

Cell viability was assayed using the CCK8 kit (CK04, Dojindo). Cells (5 × 10^3^ cells/well/100 µL) were inoculated in 96-well plates. CCK8 (10 µL/well) was added and subsequently transferred to an incubator at 37 °C with 5% CO_2_ for 4 h. The optical density (OD) value at 450 nm for each sample was measured by a microplate reader (MB-530, HEALES).

### Establishment of the xenograft PC model

Six-week-old male BALB/c nude mice were ordered from Hunan SJA Laboratory Animal Co., Ltd. LNCaP cells or LNCaP/R cells (2 × 10^8^) were injected subcutaneously into the right axilla of the mice to develop a PCa model [21]. After 14 days, the tumor size was observed and counted [21, 22], and then counted twice a week. Mice were randomly divided into 3 groups according to different experimental purposes. (1) LNCaP or LNCaP/R group (Model). (2) ECe group: Model mice were gavaged with EB-CZ aqueous decoction at a dosage of 4.94 g/kg/d (100 µL/10 g) once daily (Reference to clinical dosage). EB and CZ were each 17.29 g, cut up, dried, and decocted with water to obtain 200 mL of the concentrated solution. (3) DTX group: Model mice were injected intraperitoneally with 10 mg/kg DTX once every 7 days [21, 23, 24]. After 38 days, mice were euthanized by intraperitoneal injection of 100 mg/kg sodium pentobarbital, and tumor tissues and serum were collected. All operations involving animals were approved by the Ethics Committee of Hunan University of Chinese Medicine (LLBH-202,211,070,005).

### Cell grouping

Cells were randomly divided into 5 groups for different experimental purposes and treated for 24 h. (1) LNCaP or LNCaP/R group (Model): Cells were supplemented with RPMI-1640 medium containing 10% Model mouse serum. (2) Drug-containing serum (DCS) group: Cells were supplemented with RPMI-1640 medium containing 10% ECe mouse serum. (3) Icariin-Curcumol group: cells were supplemented with 35 µg/mL Icariin and 25 µg/mL Curcumol [12, 25, 26], both of which were ordered from Shanghai Yuanye Biotechnology Co, Ltd. (4) DTX group: Cells were supplemented with 5 nM DTX.(5) Icariin-Curcumol + DTX group: Cells were supplemented with 35 µg/mL icariin, 25 µg/mL curcumol and 5 nM DTX.

### Hematoxylin-eosin (HE) staining

The tumors were collected and subsequently embedded, dewaxed and sectioned. The tissues underwent successive staining with hematoxylin (AWI0009, Abiowell) and eosin (AWI0020, Abiowell). Sections were immersed in xylene solution, sealed with neutral gum, and then morphological changes were observed through a microscope (BA410, Motic).

### Immunohistochemistry (IHC) staining

Tumor tissue sections were placed in xylene and gradient ethanol (75-100%). Sections were immersed in 0.01 M citrate solution, followed by thermal antigen repair. After cooling, the sections were washed three times with PBS. 1% periodate was added and incubated for 10 min to inactivate the endogenous enzyme. Sections were mixed with Ki67 antibody (1:300, ab16667, Abcam) and incubated overnight at 4 °C, followed by co-incubation with secondary antibody (1:100, AWS0003, Abiowell) for 30 min. After PBS washing, DAB solution and hematoxylin were added. After dehydration by immersion in gradient alcohol (60-100%), the sections were immersed in xylene. Finally, neutral gum was used to seal the sections, and the sections were transferred to the microscope for observation and image acquisition (magnification 100× and 400×). The images were analyzed by Image-Pro-Plus software to obtain the positivity rate to measure Ki67 expression.

### Quantitative real-time PCR (qRT-PCR)

Total RNAs were extracted with TRIzol (15,596,026, Thermo), and reversely transcribed to prepare cDNAs using a cDNA synthesis kit (CW2569, CWBIO). The relative expression of targets was performed using the UltraSYBR Mixture kit (CW2601, ConWin) on QuantStudio 1 Real-Time PCR (Thermo). The following experimental parameters were applied for PCR amplification: 95 °C for 30 s, and 40 cycles of 95 °C for 5 s and 60 °C for 15 s. Targets were normalized with reference to β-actin. The primers are listed in Table 1.

**Table 1:** Primer sequences

Targets	F (5’-3’)	R (5’-3’)
AR	GCCCAGTAACTACCCGAGCAT	TCCTGATTCCCATGACCCCTT
PSA	CTGCTCGTGGGTCATTCTGA	TAGACAGGTCGGTGGGACAA
PI3K	TGCGTCTACTAAAATGCATGG	AACTGAAGGTTAATGGGTCA
Akt1	AGCCCTGGACTACCTGCACTCG	CTGTGATCTTAATGTGCCCGTCCT
mTOR	CCAAAGGCAACAAGCGATCCCGAA	CTCCAAGTTCCACACCGTCCA
HIF-1α	TGGTATTATTCAGCACGACT	GCCAGCAAAGTTAAAGCATC
c-Myc	CACTAACATCCCACGCTCTGA	AAACCGCATCCTTGTCCTGT
hnRNPs	AGACGAAGACTGAGCGGTTG	AGCCGAAAACAAGAAGGGGA
VEGF	TGCTCTACTTCCCCAAATCACT	ACTCACTTTGCCCCTGTCG
HK2	GTGAATCGGAGAGGTCCCAC	GCTAACTTCGGCCACAGGAT
PFK1	AATCTGCAAGAAAGCAGCGG	TACCAACTCGAACCACAGCC
PKM2	CGTCATTCATCCGCAAGGCAT	CACGAGCCACCATGATCCCA
β-actin	ACCCTGAAGTACCCCATCGAG	AGCACAGCCTGGATAGCAAC

### Enzyme-linked immunosorbent assay (ELISA)

The contents of glucose (A154-1-1), adenosine triphosphate (ATP, A095-1-1), lactic acid (A019-2-1), lactate dehydrogenase (LDH, A020-2-2), and pyruvate dehydrogenase (PDH, BC0385) were measured according to the manual. The glucose, ATP, lactic acid, and LDH ELISA kits were obtained from Nanjing Jiancheng Bioengineering Institute. The PDH ELISA kit was ordered from Solarbio.

### Western blot analysis

Total protein was obtained by RIPA (AWB0136, Abiowell), and the concentration was determined using a BCA kit (AWB0104, Abiowell). Subsequently, the proteins were separated by 10% SDS-PAGE and transferred to NC membranes. After blocking, the membranes were incubated with Glut1 (1:4000, 21829-1-AP, Proteintech), Glut4 (1:3000, 66846-1-Ig, Proteintech), MCT4 (1:10000, 22787-1-AP, Proteintech), and β-actin (1:5000, 66009-1-Ig, Proteintech) overnight at 4 °C. Then, membranes were incubated with HRP-labeled anti-mouse (1:5000, SA00001-1, Proteintech) and anti-rabbit (1:6000, SA00001-2, Proteintech) at room temperature for 90 min. Finally, membranes were transferred to ECL Plus luminescent solution (AWB0005, Aiowell) for 1 min, and the protein bands were visualized by a gel imaging system (ChemiScope6100, CLiNX).

### High-performance liquid chromatography-mass spectrometry (HPLC-MS)

The EB-CZ aqueous decoction was filtered and dried using a freeze drier. The dried powder was mixed with methanol and extracted by sonication, followed by filtration through a 0. 45 μm microporous filter membrane. Liquid chromatographic separation and mass spectrometric detection were performed using an AB TripleTOF® 5600 + LC/MS system. The chromatographic separation was performed on a Waters HSS T3 column (100 × 2.1 mm, 1.7 μm). The column temperature was 40℃. The mobile phase consisted of water containing 0.1% formic acid (A) and acetonitrile (B). The gradient elution adjustments were set as follows: 0-1.5 min, 1% B; 1.5–16.5 min, 99% B; 16.5–20 min, 1% B, with a flow rate of 0.3 mL/min. The injection volume was 3 µL. MS spectra were obtained using positive and negative ion modes. Mass condition was adjusted as follows: TOF, 60-1250 m/z; ion source gas, 55 psi; curtain gas, 35 psi; temperature, 550 °C; DP, 80 V; CE, 30 V; ionSpray voltage, 5500 V.

### Molecular docking

The protein structures of Akt1 and the 3D structures of icariin and curcumol were obtained from the PDB and PubChem databases, respectively. The binding of icariin and curcumol to Akt1 was studied using Autodock Vina software. The main operations include removing water molecules, adding non-polar hydrogen, calculating Gasteiger charges, assigning AD4 types, adjusting the total charge number of ligand molecules, and selecting ligand-twistable bonds. Visual analysis was performed using Discovery Studio.

### Apoptosis analysis by flow cytometry

Apoptosis was detected according to the instructions of the apoptosis kit (KGA1030, KeyGEN BioTECH). Briefly, cells were digested with EDTA-free trypsin and washed with PBS to a concentration of 3.2 × 10^5^ cells. Cells were suspended by adding 500 µL of Binding buffer. Subsequently, the percentage of apoptotic cells was assessed by flow cytometry (A00-1-1102, Beckman).

### Statistical analysis

Statistical analysis was performed using GraphPad Prism 9.0 software. All data were presented as the mean ± standard deviation values. An unpaired t-test was used to evaluate the differences between the two groups. One-way analysis of variance (ANOVA) was used to measure differences between multiple groups. P < 0.05 indicates a significant difference.

## Results

### Determination of ingredients-targets-PCa network

**Fig. 1:**
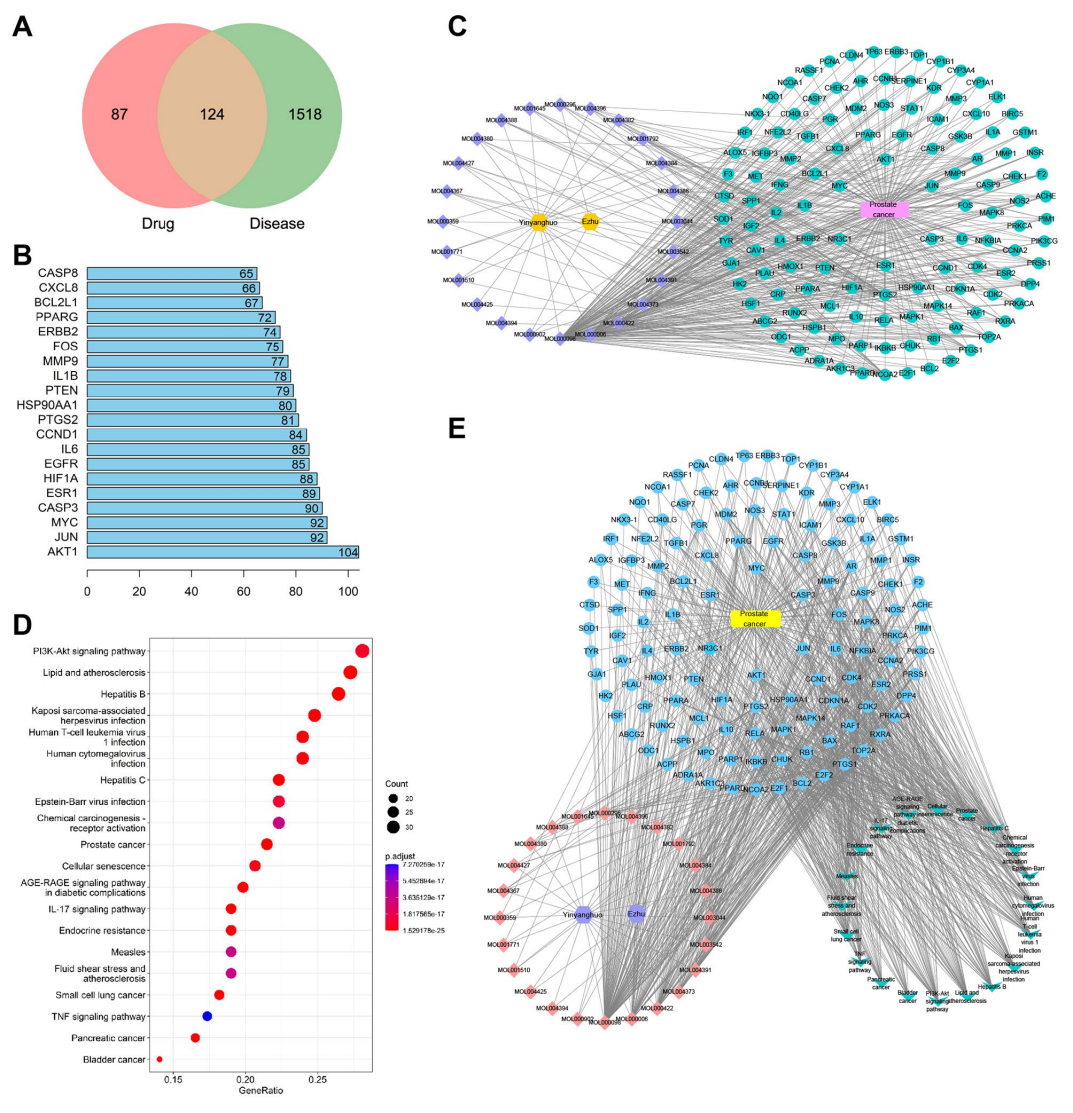
Network pharmacological analysis between EB-CZ ingredients and PCa. **(A)** Venn diagram of the co-regulatory targets between ECe and PCa. **(B)** The degree of 20 core targets of the PPI network. Higher degree value indicates a closer relationship. **(C)** EB-CZ ingredients-targets-PCa network. Yellow dot represents the drug. Purple squares represent EB-CZ ingredients. Green dots represent target genes associated with PCa. **(D)** KEGG enrichment analysis of 20 core signaling pathways. **(E)** EB-CZ ingredients-targets-signaling pathways-PCa network. Purple hexagons represent the drug. Pink squares represent ingredients. Blue dots represent targets associated with PCa. Green arrows represent signaling pathways

We applied pharmacological network analysis to find the anti-PCa targets of EB-CZ active ingredients. A search from the TCMSP database obtained 23 compounds and 208 targets in EB, and 4 compounds and 21 targets were found in CZ (Table 2). A total of 1642 PC-related target genes were obtained from GeneCards, NCBI and OMIM. As shown in Fig. 1A and 124 common target genes existed between ECe and PCa for subsequent analysis. Then, we constructed the PPI network and performed topological analysis to obtain the top 20 highly valued target genes, among which Akt1 had the largest degree values (degree = 104) (Fig. 1B). Visualization of the network diagram of EB-CZ active ingredients and their potential targets with PCa suggested that these compounds and target genes may be important nodes for anti-PCa (Fig. 1C). KEGG enrichment was performed based on common targets to elucidate the potential molecular pathways of EB-CZ active ingredients in regulating PCa. A total of 163 signaling pathways were obtained, and the 20 most closely related pathways were presented, including the phosphoinositide 3-kinase (PI3K)-Akt signaling pathway, lipid and atherosclerosis and hepatitis B (Fig. 1D). Subsequently, we constructed an EB-CZ ingredients-target-functional pathway-PCa network diagram to visually present the interactions between EB-CZ active components and PCa (Fig. 1E). In short, these results suggested that the EB-CZ ingredients regulated the PCa progression probably through the PI3K-Akt signaling pathway.

**Table 2:** Potential active components of EB-CE.

Mol ID	Molecule name	OB (%)	DL	MW	Herb name
MOL000296	hederagenin	36.91	0.75	414.79	*Curcuma zedoaria*
MOL000906	wenjine	47.93	0.27	282.37	*Curcuma zedoaria*
MOL000940	bisdemethoxycurcumin	77.38	0.26	308.35	*Curcuma zedoaria*
**MOL000902**	**curcumol**	103.55	0.13	236.39	*Curcuma zedoaria*
MOL001510	24-epicampesterol	37.58	0.71	400.76	*Epimedium brevicornu*
MOL001645	Linoleyl acetate	42.10	0.20	308.56	*Epimedium brevicornu*
MOL001771	poriferast-5-en-3beta-ol	36.91	0.75	414.79	*Epimedium brevicornu*
MOL001792	DFV	32.76	0.18	256.27	*Epimedium brevicornu*
MOL003044	Chryseriol	35.85	0.27	300.28	*Epimedium brevicornu*
MOL003542	8-Isopentenyl-kaempferol	38.04	0.39	354.38	*Epimedium brevicornu*
MOL000359	sitosterol	36.91	0.75	414.79	*Epimedium brevicornu*
MOL000422	kaempferol	41.88	0.24	286.25	*Epimedium brevicornu*
MOL004367	olivil	62.23	0.41	376.44	*Epimedium brevicornu*
MOL004373	Anhydroicaritin	45.41	0.44	368.41	*Epimedium brevicornu*
MOL004380	C-Homoerythrinan, 1,6-didehydro-3,15,16-trimethoxy-, (3.beta.)-	39.14	0.49	329.48	*Epimedium brevicornu*
MOL004382	Yinyanghuo A	56.96	0.77	420.49	*Epimedium brevicornu*
MOL004384	Yinyanghuo C	45.67	0.50	336.36	*Epimedium brevicornu*
MOL004386	Yinyanghuo E	51.63	0.55	352.36	*Epimedium brevicornu*
MOL004388	6-hydroxy-11,12-dimethoxy-2,2-dimethyl-1,8-dioxo-2,3,4,8-tetrahydro-1 H-isochromeno[3,4-h]isoquinolin-2-ium	60.64	0.66	370.41	*Epimedium brevicornu*
MOL004391	8-(3-methylbut-2-enyl)-2-phenyl-chromone	48.54	0.25	290.38	*Epimedium brevicornu*
MOL004394	Anhydroicaritin-3-O-alpha-L-rhamnoside	41.58	0.61	676.73	*Epimedium brevicornu*
MOL004396	1,2-bis(4-hydroxy-3-methoxyphenyl)propan-1,3-diol	52.31	0.22	320.37	*Epimedium brevicornu*
MOL004425	Icariin	41.58	0.61	676.73	*Epimedium brevicornu*
MOL004427	Icariside A7	31.91	0.86	462.49	*Epimedium brevicornu*
MOL000006	luteolin	36.16	0.25	286.25	*Epimedium brevicornu*
MOL000622	Magnograndiolide	63.71	0.19	266.37	*Epimedium brevicornu*
MOL000098	quercetin	46.43	0.28	302.25	*Epimedium brevicornu*

### ECe alleviates PC development through the PI3K-Akt signaling pathway

**Fig. 2:**
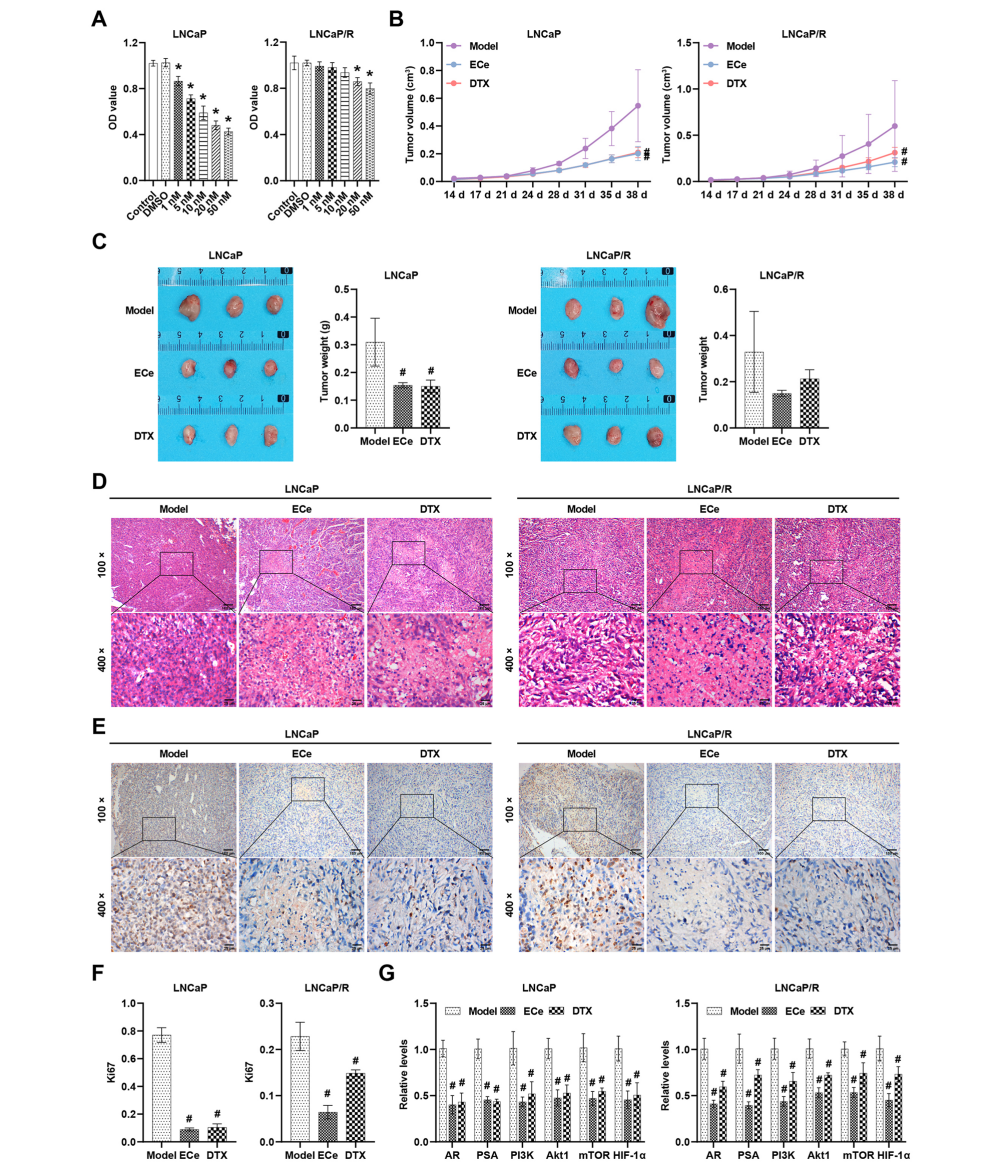
ECe and DTX inhibit PCa development by impeding PI3K-Akt signaling. **(A)** Determination of cell viability by CCK8 assay. **(B)** Documentation of xenograft tumor size during modeling. **(C)** After the nude mice were sacrificed, the weight of the xenograft tumors was recorded. **(D)** Representative HE staining images of tumor tissues. **(E)** Representative IHC staining images of Ki67 in tumor tissues. **(F)** Statistical analysis of Ki67 expression. **(G)** The relative levels of AR, PSA, PI3K, Akt1, mTOR, and HIF-1α were determined. *P < 0.05 compared with the DMSO group; #*P* < 0.05 compared with the Model group

To study the effect of ECe on DTX resistance in PCa, xenograft models of LNCaP or LNCaP/R cells were developed. The effects of different doses (1 nM, 5 nM, 10 nM, 20 nM, and 50 nM) of DTX on LNCaP and LNCaP/R cells were investigated. Compared with the DMSO group, different concentrations of DTX decreased LNCaP cell viability, and only 20 and 50 nM DTX inhibited LNCaP/R cell viability (Fig. 2A). Therefore, our developed model of LNCaP cells resistant to DTX (LNCaP/R) was feasible. ECe and DTX reduced the size and weight of xenograft tumors compared to the Model group (Fig. 2B C), suggesting that ECe and DTX exerted anti-PCa effects. HE staining observed that ECe and DTX alleviated inflammatory cell infiltration in xenograft tumors compared to the Model group (Fig. 2D). In addition, Ki67 expression was reduced in the ECe or DTX group (Fig. 2E F). The potential involvement of the PI3K-Akt pathway was further elaborated. ECe and DTX inhibited the levels of AR, PSA, PI3K, Akt1, mammalian target of rapamycin (mTOR) and hypoxia-inducible factor-1α (HIF-1α) compared to the Model group (Fig. 2G). Our data illustrated that ECe and DTX interfered with AR activity and the PI3K-Akt pathway to inhibit the development of PCa and that ECe alleviated DTX resistance.

### ECe regulates the Warburg effect in PC animal models

**Fig. 3:**
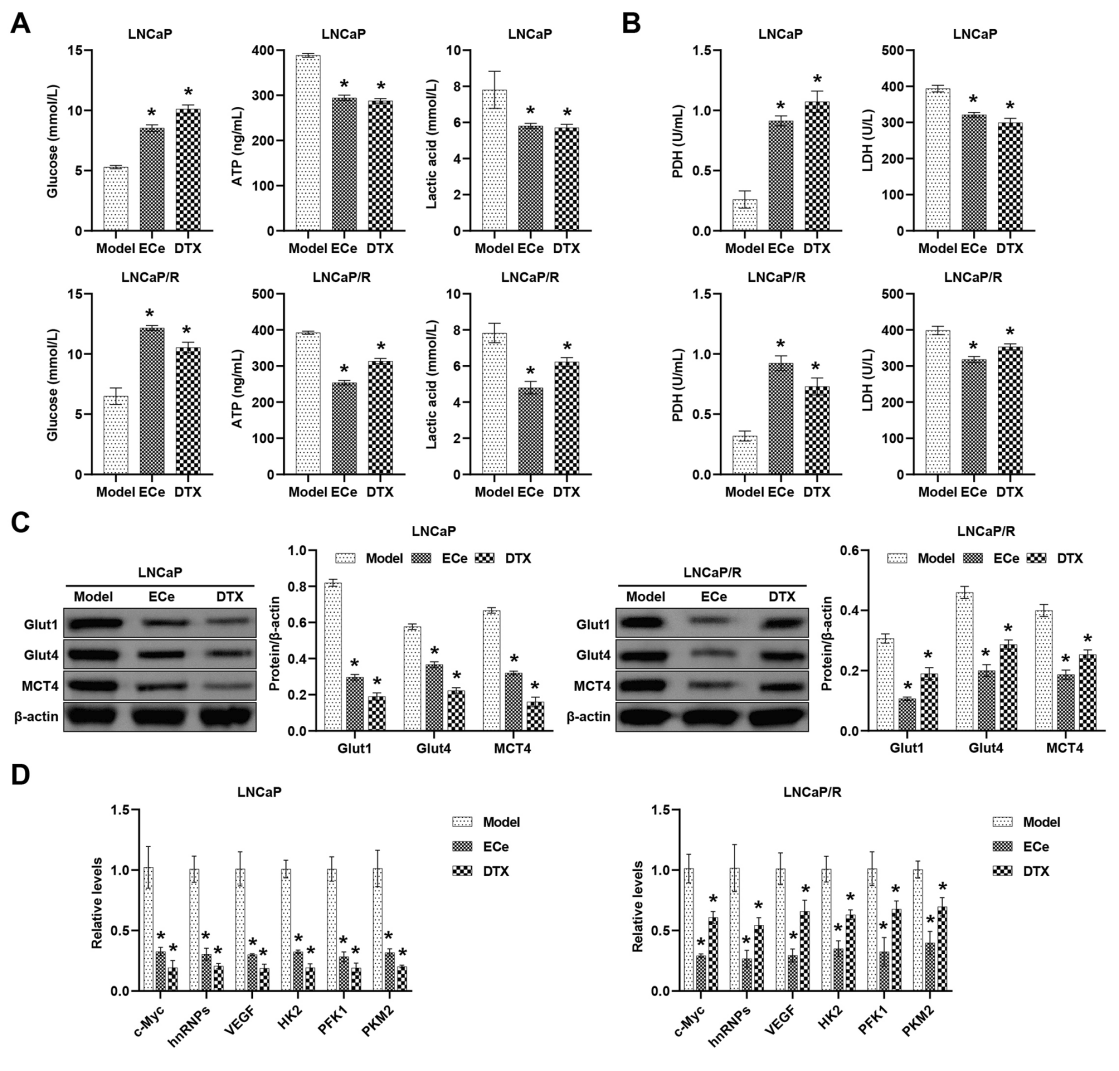
ECe and DTX regulate the Warburg effect. **(A)** Determination of glucose, ATP and lactic acid content. **(B)** Determination of PDH and LDH levels. **(C)** The protein abundance of Glut1, Glut4, and MCT4 was examined. **(D)** The relative levels of c-Myc, hnRNPs, VEGF, HK2, PFK1, and PKM2 were detected. *P < 0.05 compared with the Model group

The Warburg effect, also defined as “aerobic glycolysis”, reflects the metabolic program during cancer development [27]. Compared to the Model group, ECe and DTX increased extracellular glucose and decreased lactic acid and ATP production (Fig. 3A). ELISA showed that ECe and DTX decreased LDH levels and increased PDH (Fig. 3B). In addition, the ECe or DTX group exhibited reduced protein abundance of Glut1, Glut4 and MCT4 compared to the Model group (Fig. 3C). c-Myc, heterogeneous nuclear ribonucleoproteins (hnRNPs), vascular endothelial-derived growth factor (VEGF), phosphofructokinase 1 (PFK1) and pyruvate kinase M2 (PKM2) levels were significantly reduced in the ECe or DTX group compared to the Model group (Fig. 3D). These results elaborated that ECe and DTX had important roles in regulating the Warburg effect in PC development.

### Icariin and curcumol can dock with AKT1

**Fig. 4:**
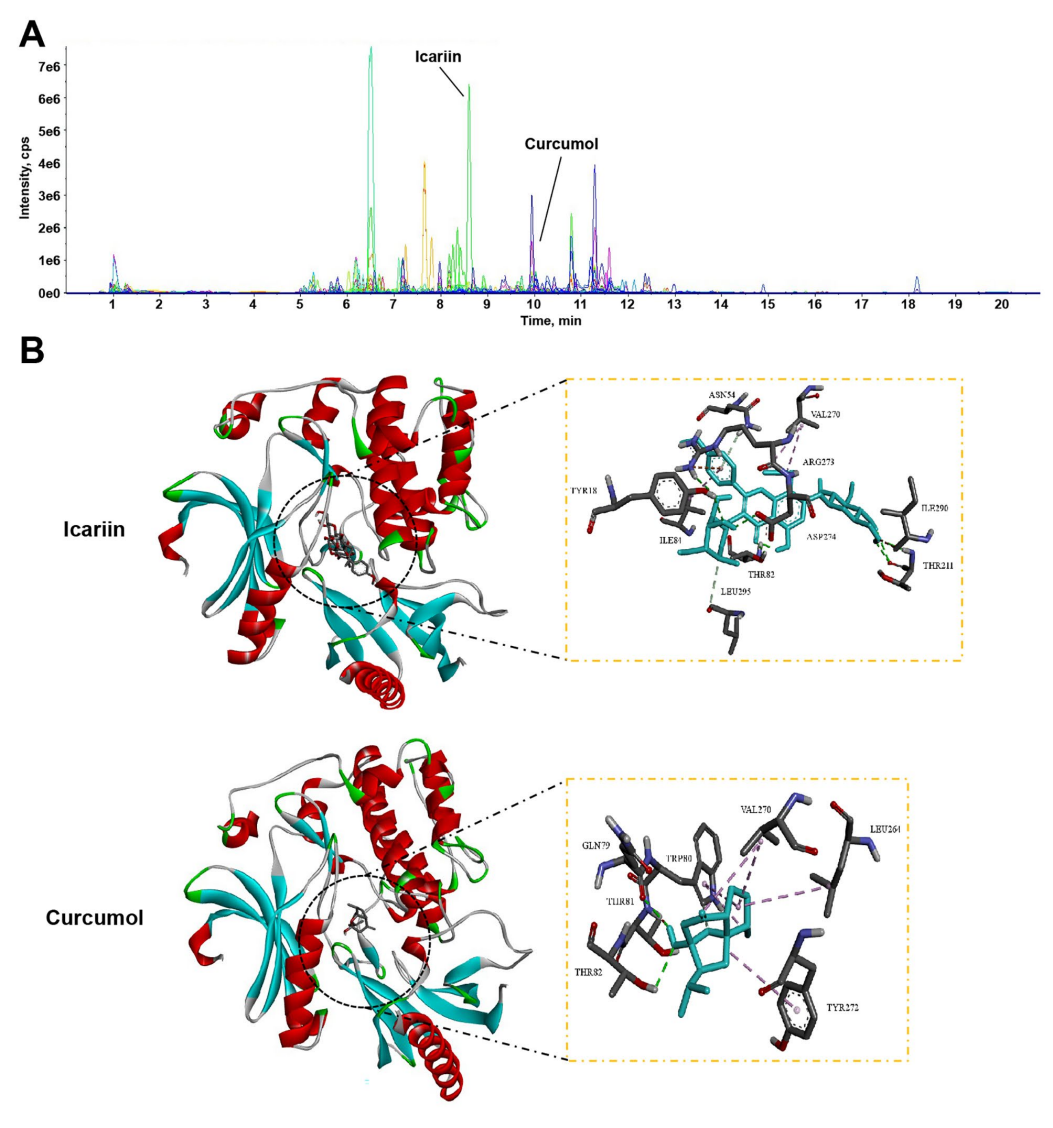
Icariin and curcumol can dock with AKT1. **(A)** The representative LC-MS chromatograms of ECe in negative and positive modes. **(B)** Molecular docking of icariin or curcumol and Akt1

Icariin and curcumol were identified from the aqueous decoction by HPLC-MS analysis (Fig. 4A; Table 3). Therefore, we speculated that icariin and curcumol might be the active components of ECe exerting anti-PC effects. Further molecular docking analysis revealed that icariin and curcumol could dock with the protein structure of Akt1 (Fig. 4B), indicating that icariin and curcumol interacted with AKT1. Therefore, we selected icariin and curcumol for the follow-up study.

**Table 3:** Identification of Icariin and Curcumol from ECe in positive and negative modes

Expected RT	Area	Height	Retention Time	Adduct / Charge	Formula	Precursor Mass	Fragment Mass	Component Name	Mass Error Confidence	Isotope Confidence	Library Confidence	Found At Mass	Mass Error (ppm)	Library Hit	Library Score	Isotope Ratio Difference
11.88	1378000	386900	11.88	[M + H]+	C15H24O2	237.185	N/A	Curcumol (11.877)	Green	Green	Green	237.1851	1	Curcumol	99	1.5
10.87	516400	144700	10.87	[M + H]+	C15H24O2	237.185	N/A	Curcumol (10.873)	Green	Green	Green	237.185	0.5	Curcumol	73.9	2.4
9.96	281200	34540	9.97	[M + H]+	C15H24O2	237.185	N/A	Curcumol (9.957)	Green	Yellow	Green	237.1845	-1.8	Curcumol	77.7	15.8
7.35	238900	37370	7.35	[M + H]+	C15H24O2	237.185	N/A	Curcumol (7.347)	Green	Yellow	Yellow	237.1848	-0.3	Curcumol	47.5	6.3
8.6	31090000	6420000	8.6	[M + H]+	C33H40O15	677.244	N/A	Icarrin (8.602)	Green	Yellow	Green	677.243	-1.4	Icarrin	51.1	15
10.02	2497000	652100	10.02	[M + H]+	C33H40O15	677.244	N/A	Icarrin (10.022)	Green	Green	Green	677.2438	-0.3	Icarrin	71.8	1.7
9.29	1985000	512900	8.91	[M + H]+	C33H40O15	677.244	N/A	Icarrin (9.294)	Green	Green	Green	677.2435	-0.8	Icarrin	95.9	4
10.66	186200	20720	10.45	[M + H]+	C33H40O15	677.244	N/A	Icarrin (10.660)	Green	Green	Green	677.2435	-0.8	Icarrin	86.1	0.6
8.01	1808000	237800	8.38	[M + H]+	C33H40O15	677.244	N/A	Icarrin (8.014)	Green	Yellow	Green	677.2434	-0.9	Icarrin	97.7	5.1
8.79	2984000	752700	8.59	[M-H]-	C33H40O15.HCOOH	721.235	N/A	Icarrin + HCOOH (8.790)	Green	Red	Green	721.2234	-16	Icarrin + HCOOH	99.3	Infinity
8.27	2984000	752700	8.59	[M-H]-	C33H40O15.HCOOH	721.235	N/A	Icarrin + HCOOH (8.267)	Green	Red	Green	721.2234	-16	Icarrin + HCOOH	99.3	Infinity
12.17	11430	1399	11.96	[M-H]-	C33H40O15.HCOOH	721.235	N/A	Icarrin + HCOOH (12.170)	Green	Green	Green	721.2273	-10.6	Icarrin + HCOOH	85.8	4.6

### Icariin-Curcumol reserves DTX resistance via the PI3K-AKT signaling pathway and Warburg effect in vitro

**Fig. 5:**
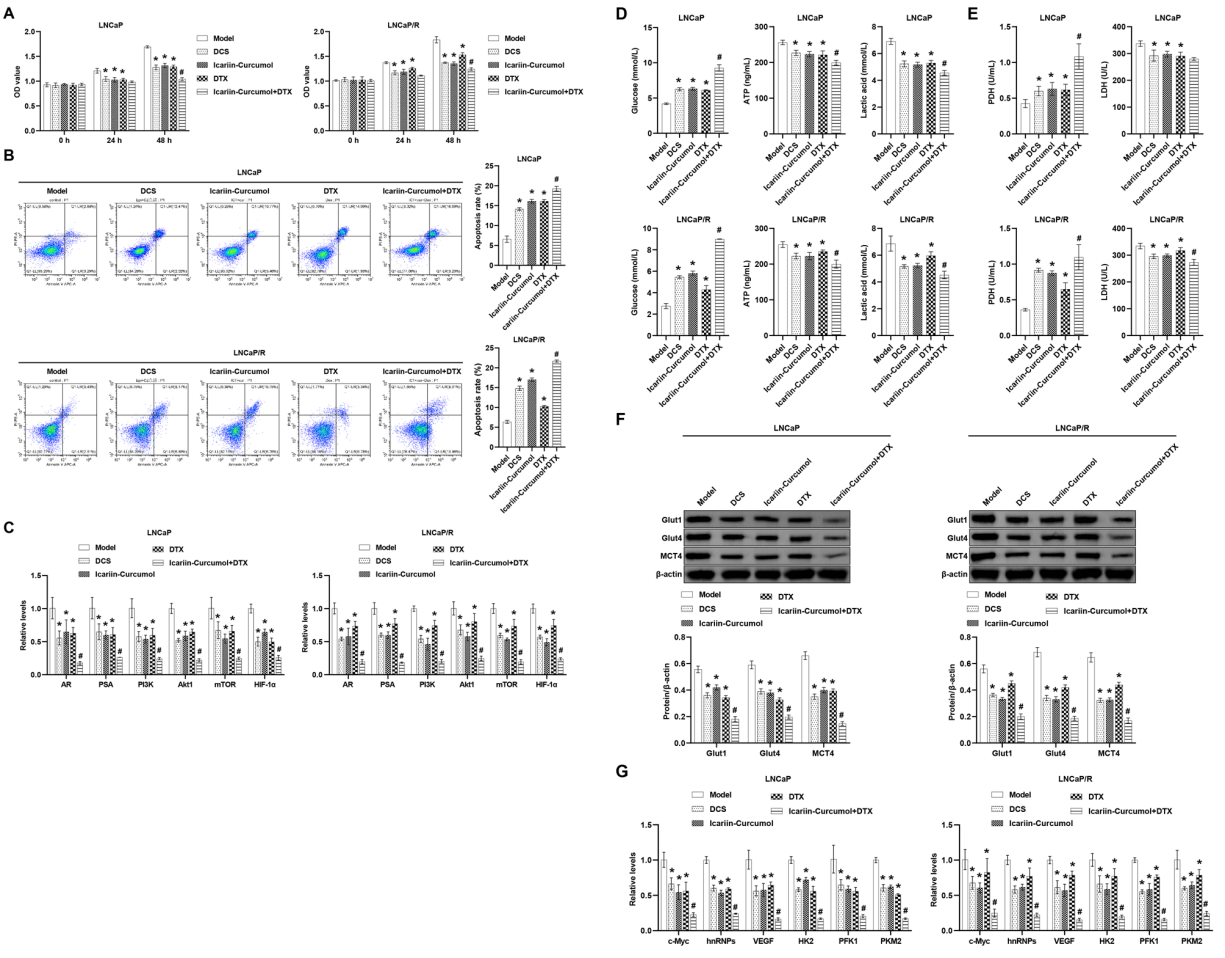
Icariin-Curcumol attenuates DTX resistance by regulating the PI3K-Akt signaling pathway and Warburg effect. **(A)** Determination of cell viability at 0, 24, and 48 h by CCK8 assay. **(B)** Analysis of apoptosis by flow cytometry. **(C)** The relative levels of AR, PSA, PI3K, Akt1, mTOR, and HIF-1α were determined. **(D)** Determination of glucose, lactate and ATP content. **(E)** Determination of PDH and LDH levels. **(F)** The protein abundance of Glut1, Glut4, and MCT4 was examined. **(G)** The relative levels of c-Myc, hnRNPs, VEGF, HK2, PFK1, and PKM2 were detected. *P < 0.05 compared with the Model group; #*P* < 0.05 compared with the DTX group

The function of icariin and curcumol in DTX resistance in vitro was then discussed. DCS, Icariin-Curcumol and DTX decreased cell viability compared to the Model group (Fig. 5A). DCS, Icariin-Curcumol and DTX promoted apoptosis (Fig. 5B). The DCS, Icariin-Curcumol and DTX groups exhibited reduced levels of AR, PSA, PI3K, Akt1, mTOR and HIF-1ɑ (Fig. 5C). In addition, DCS, Icariin-Curcumol and DTX increased extracellular glucose and PDH and decreased lactic acid, ATP and LDH levels compared to the Model group (Fig. 5D and E). WB analysis showed that DCS, Icariin-Curcumol and DTX decreased the protein abundance of Glut1, Glut4 and MCT4 (Fig. 5F). c-Myc, hnRNPs, VEGF, PFK1 and PKM2 levels were significantly reduced in the DCS, Icariin-Curcumol and DTX groups compared to the Model group (Fig. 5G). Notably, the combination of Icariin-Curcumol and DTX further enhanced the effect of the separate agents (Fig. 5). Thus, our evidence revealed that Icariin-Curcumol attenuated DTX resistance through modulation of the PI3K-Akt pathway and the Warburg effect and that Icariin-Curcumol and DTX have synergistic effects.

## Discussion

In recent years, anti-androgen therapy has shown an exciting performance in improving the adverse outcomes of CRPC [28]. Among these, DTX is recommended as a first-line option for androgen deprivation therapy [29]. Unfortunately, despite the improvement in overall survival, clinical symptoms and pathological phenotype of DTX in CRPC patients, the emergence of DTX resistance has inevitably limited its efficacy [30]. Accumulating reports support the reversal of phytochemicals in DTX resistance [31]. For example, Quercetin inhibits DTX resistance via the AR and PI3K/AKT pathways in drug-resistant PCa cells and animal models [21]. Artesunate limited the growth behavior of drug-resistant PCa cells [32]. In this study, we demonstrated that ECe and DTX effectively promoted tumor regression based on the “multi-component, multi-pathway, multi-target” theory. For the first time, we reported the function of icariin and curcumol in combating DTX resistance in PCa. Further studies revealed the synergistic effect of Icariin-Curcumol and DTX. The underlying mechanism involved PI3K/Akt signaling pathway and the Warburg effect.

Currently, network pharmacology has been widely used in the field of transitional Chinese medicine (TCM), which can predictively establish “active ingredient-protein/gene-disease” networks, providing an effective paradigm for a more comprehensive insight into disease modules and precise interventions [33, 34]. We analyzed the EB-CZ active ingredient-signaling pathway-PCa network and found that icariin and curcumol were included in the EB-CZ active ingredient. In addition, icariin and curcumol were similarly detected in ECe using HPLC-MS. Among the targets of EB-CZ active components acting on PCa, Akt1, JUN, MYC, CASP3 and ESR1 were the core targets. KEGG analysis showed that the functional signals of EB-CZ active components in anti-PCa were mainly focused on the PI3K-Akt signaling pathway, lipid and atherosclerosis, hepatitis B and others. These results tentatively suggested the possibility of icariin and curcumol in resisting PCa development.

AR signaling reactivation is thought to contribute to the development of DTX resistance [35]. Androgens, particularly testosterone and dihydrotestosterone, stimulate PC cell proliferation and inhibit apoptosis. There is evidence that testosterone supplementation impairs the reduced antitumor activity of DTX and that AR activation reverses the tumor regression of DTX treatment [36]. In our study, ECe, Icariin-Curcumol and DTX contributed to reducing the expression of AR and its downstream protein PSA in the LNCaP/R model. Icariin-Curcumol effectively impeded LNCaP/R cell proliferation and promoted apoptosis, and ECe and Icariin-Curcumol had superior therapeutic effects than DTX. The effects of ECe and Icariin-Curcumol in the LNCaP model were close to those of DTX. In addition, the combination of Icariin-Curcumol and DTX further enhanced the effects of both alone, suggesting the synergistic effect of Icariin-Curcumol and DTX.

Upregulation of PI3K-Akt signaling has been suggested as an additional reason for the enhanced drug resistance of DTX [37, 38]. DTX has been shown to inhibit PI3K/Akt phosphorylation [39]. Silencing of CNTN-1 has been demonstrated to improve proliferation and inhibit epithelial-mesenchymal transition in PC3/R and DU145/R cells by inhibiting PI3K/Akt signaling [40]. Additionally, solamargine has been reported to inhibit PI3K/Akt phosphorylation and synergize with DTX in inhibiting tumor growth. The application of myristoylated Akt (Myr-Akt) partially counteracted the inhibitory effects of solamargine on CRPC cell deterioration [38]. DTX effectively inhibited the PI3K/Akt signaling pathway [41]. In this study, we observed that ECe, Icariin-Curcumol and DTX hindered the expression of PI3K, Akt1, and downstream factors mTOR and HIF-1ɑ in LNCaP/R and LNCaP models. Molecular docking showed that the core target Akt1 could dock with icariin and curcumol, respectively. Similarly, the regulation of the abundance of these factors by ECe and Icariin-Curcumol was superior to that of DTX in LNCaP/R without significant differences in LNCaP. These results suggested that Icariin-Curcumol inhibited the PI3K-Akt pathway to reverse DTX resistance. In previous reports, PI3K-Akt signaling can treat PC resistance in an AR-dependent or independent manner [42]. The interaction between AR signaling and PI3K/Akt signaling pathway was shown in PC [43]. However, the link between AR and PI3K/Akt signaling pathway deserves further exploration.

Interestingly, we observed that ECe and Icariin-Curcumol appear to counteract DTX resistance by modulating the Warburg effect in LNCaP/R and LNCaP cells. The increased rate of glycolysis is considered a common metabolic change in cancer [44]. Enzymes involved in glucose metabolism have been found to be dysregulated in PCa, and the reduction in the Warburg effect has been shown to accompany inhibition of PCa xenograft tumor growth and drug resistance [45, 46]. Inhibition of glycolysis has been suggested as a potential strategy to overcome cancer drug resistance [47]. DTX has been reported to inhibit PCa cell proliferation and the Warburg effect by targeting the Smad3/HIF-1α signaling pathway [48]. Furthermore, previous research has found that Zhoushi Qi Ling decoction downregulates the levels of lncRNA SNHG10 in PCa cells, and overexpression of SNHG10 reversed the effect of the decoction on cell proliferation and glycolysis in CRPC cells [49]. Overexpression of SNHG10 also promoted glucose depletion and lactate release and enhanced glycolysis in CRPC cells [49]. In this study, we found that ECe, Icariin-Curcumol and DTX increased extracellular glucose and decreased lactic acid and ATP production. Moreover, ECe, Icariin-Curcumol, and DTX groups showed reduced LDH and increased PDH. ECe, Icariin-Curcumol and DTX blocked the glycolytic transport proteins (Glut1 and Glut4), the lactic acid transport carrier MCT4, and the accumulation of the glycolytic rate-determining enzymes PFK1 and PKM2. It was found that activation of the PI3K/Akt signaling pathway plays an important role in cancer cells [50]. PI3K/Akt contributes to the rapid transport and consumption of glucose for ATP and lactic acid production in drug-resistant cells by upregulating the expression of glycolysis-related enzymes, such as the Glut family [51, 52]. Therefore, we propose a reasonable hypothesis that Icariin-Curcumol inhibits PI3K/Akt signaling pathway to hinder glycolysis in PCa cells, which leads to ATP depletion and ultimately reverses DTX resistance. This points the direction for our subsequent studies.

The present study still has some limitations. It is necessary to obtain more direct in vivo evidence to support the anti-PCa effect of Icariin-Curcumol, including considerations of the structural stability and bioavailability of the drug monomers. Additionally, different PCa models may impact the therapeutic effect of ECe. Furthermore, due to limitations in funding and time, we were unable to provide certain evidence, such as the effect of ECe/Icariin-Curcumol on the phosphorylation levels of PI3K and Akt, as well as downstream substrates like mTOR and HIF-1α. Similarly, we did not evaluate the effects of PI3K activators or other overexpression reagents on Icariin-Curcumol-regulated DTX resistance. In addition to measuring the production of key enzymes involved in the glycolytic pathway, it would be beneficial to incorporate strategies such as Seahorse energy metabolism analysis. There are also several aspects that require further investigation. Firstly, the specific efficacy of the combination therapy of Icariin-Curcumol and DTX in the clinical setting needs to be determined. Additionally, since Icariin has multiple metabolites such as icariside I, icariside II, icaritin, and desmethylicaritin [53], it remains to be established whether the effects of Icariin are mediated through its specific products. Similarly, the metabolites of curcumol have been less studied, and it is possible that they also play a role in DTX resistance. Mechanistically, it is unclear whether overexpression of Glut1, Glut4, or MCT4 impedes the inhibitory effect of Icariin-Curcumol on DTX resistance. Furthermore, glucose metabolism is closely linked to mitochondrial oxidative stress, as mitochondria are the primary site of sugar and ATP production in eukaryotes [54]. Therefore, exploring the effect of Icariin-Curcumol on oxidative stress may provide insights into the role of glycolysis in DTX resistance. Moreover, AR has been found to induce the expression of Glut1 and MCT4 [55]. Activated Akt has been shown to stimulate glucose uptake, promote Glut1 expression, and enhance glycolysis [47, 56]. AR signaling is closely associated with PI3K/Akt/mTOR signaling pathway [43]. However, the crosstalk between AR-PI3K/Akt-glycolysis pathways in the combination therapy of Icariin-Curcumol and DTX remains unclear. Moreover, autophagy has been reported to regulate the sensitivity of DTX in LNCaP, PC3, and DU145 cells during combination therapy [57]. However, the role of ECe in the autophagic mechanism remains to be revealed. These questions deserve to be addressed in future studies.

## Conclusion

In conclusion, our study confirmed the therapeutic effect of Icariin-Curcumol on DTX resistance and the synergistic effect of Icariin-Curcumol and DTX in treating PCa. Mechanistic studies showed that Icariin-Curcumol enhanced chemosensitivity by inhibiting the PI3K/Akt signaling pathway and the Warburg effect. Our study broadened the application of TCM theory in the treatment of PCa and suggested that Icariin-Curcumol has the potential as an effective target for treating DTX resistance.

## Data Availability

The data used to support the findings of this study are included within the article.
